# Supplementary material for: Recognition of food images based on transfer learning and ensemble learning
Source: PLoS One. 2024 Jan 19;19(1):e0296789. doi: 10.1371/journal.pone.0296789 (PMC10798480; doi:10.1371/journal.pone.0296789)
Supplement: Attachment — Submitted filename: PLOSone Revised report_14 Sep23.pdf [file pone.0296789.s002.pdf]

Authors compared transfer learning from VGG19, ResNet50, MobinetV2, and AlexNet. Then, fine-tune them by freezing certain layers and retraining the remaining layers of food images. Later, the outputs from these fine-tuned models were ensembled using weight-ensemble and vote-ensemble techniques. They concluded that weighed ensemble yields the highest accuracy of 96.88%.

Ensemble learning is a well-established technique in machine learning and has been an active area of research for many years. Therefore, the novelty of the proposed ensemble learning approach as raised by Reviewers 1 and 3 too may depend on the specific criteria set by the editor for publication. If the revised paper is accepted for publication, there are some concerns that need to be addressed as follows:

1. Figure 1 was removed in the revised manuscript without track changes, but it remains in version with track changes. Which one is the correct version?
2. In the with track changes manuscript, the revised Stage 1 in Figure 2 is misleading. It currently shows transfer learning from source dataset to target dataset, followed by data preprocessing. It should be revised to reflect the correct order: data preprocessing of the dataset, followed by transfer learning. Additionally, the figure does not align with the text explanation: "In the data preprocessing stage, the preprocessing operation of food images was mainly divided into 2 steps: data enhancement and data standardization." Please ensure that the text and figure match.
3. To improve clarity, please mention in text, Stage 1 represents preprocessing, Stage 2 is the stage of pre-training network and fine-tuning the pre-training, and Stage is Ensemble stage. Stage 2 should include 4 pre-trained models up to probability distribution, Stage 4 starts from Ensemble stage.
4. Please make sure that Stage 2 includes information up to Probability Distribution. Add in $h_i(x)$ for each Probability Distribution and H(x) at the Output.
5. Comment number 1 from Reviewer 6 should be added in the comparison of your results with other Food Image classification results in a table form in the Results section, not as the author claimed, already mentioned In Introduction part.
6. Comment number 4 from reviewer 6 has not been addressed at all, despite the authors' claim that it has been mentioned in Section 4.1.
7. Please list the six augmentation strategies in the revised manuscript.
8. The explanation of "The FPS indicator represents the time taken by the model to detect an image or the number of images processed in one second." is misleading. If we use time taken by the model to detect an image, then the smaller FTS will be a good indicator. However, if we use "the number of images processed in one second " then a larger FPS value is will be a good indicator. This leads to contradictory values for a good indicator. Please clarify this point.
9. The response to why the choice of the 4 pretrained models in Reviewers 3 and 5 should address why these four were chosen over others, not addressing the issue of limited images.
