# Supplementary material for: Recognition of food images based on transfer learning and ensemble learning
Source: PLoS One. 2024 Jan 19;19(1):e0296789. doi: 10.1371/journal.pone.0296789 (PMC10798480; doi:10.1371/journal.pone.0296789)
Supplement: Attachment — Submitted filename: Response to Reviewers.docx [file pone.0296789.s001.docx]

Response to Reviewer 1 Comments

**Point 1:** Authors have applied existing techniques such as 4 pretrained models and ensemble technique in recognition of food images. Authors are suggested to develop some innovative techniques that address critical challenges in this area. Current version of paper is missing novelty.

**Response 1:**

The Transfer learning strategy used in this paper is freezing and training. When training four Convolutional neural network models, freeze part of the feature layers, retrain the rest of the feature layers and the full connection layer, and constantly adjust the strategy according to the performance on the food data set to obtain better results.

In this paper, a food image recognition method based on ensemble learning and transfer learning was proposed to detect nutritional contents in common foods. Six strategies were used on food-11 dataset for data augmentation and a large number of comparative experiments were carried out for food recogniton. The results of four CNN models on the Food-11 dataset demonstrated that the transfer learning method could effectively reduce the overfitting phenomenon and improve the generalization performance of the model. By combining different learning strategies, it could be proven that the ensemble learning method effectively improved the accuracy of the model, and the weight-ensemble method was the best for ensemble learning, achieving an accuracy rate of 96.88%. This demonstrated that the use of deep convolution neural network could make full use of the high-level semantic features of images, and the recognition method based on ensemble learning and transfer learning could help the model learn more comprehensive and rich feature information, greatly improving the generalization performance of the model. Therefore, the method proposed in this paper is suitable for food image recognition under complex background.

The next step of research will address the issue of reduced recognition speed caused by integration methods, streamline the structure of the integration model, optimize the integration methods, and achieve faster food image recognition speed.

Response to Reviewer 2 Comments

**Point 1:** The authors claim that data augmentation strategies used mainly include six strategies. It is unclear if other techniques were also used. Were these data pre-processing techniques used in the Food-11 dataset? Is the modified dataset used for the experiments available?

**Response 1:** We used six data augmentation strategies to process the food-11 dataset, and the experimental results shown in Section 4 demonstrated the feasibility of the modified dataset. We have added relevant descriptions in the conclusion section.

**Point 2:** Other recent work that the authors may cite, stating the difference from their work:

G. Ciocca, G. Micali and P. Napoletano, "State Recognition of Food Images Using Deep Features," in IEEE Access, vol. 8, pp. 32003-32017, 2020, doi: 10.1109/ACCESS.2020.2973704.

Peihua Ma, Chun Pong Lau, Ning Yu, An Li, Jiping Sheng, Application of deep learning for image-based Chinese market food nutrients estimation, Food Chemistry, Volume 373, Part B, 2022.

**Response 2:** The first paper above combined deep features extracted from neural networks with support vector machines (SVM) as an alternative method for end-to-end classification. The second paper above optimized the Inception-v3 network using other state-of-the-art deep convolutional neural networks, achieving up to 78 % and 94 % for top-1 and top-5 accuracy, respectively.

Our work is aimed at using different convolutional neural network models for transfer learning and building integrated models using different integrated learning strategies to achieve high accuracy on the food dataset.

Response to Reviewer 3 Comments

**Point 1:** Four deep networks are used for extracting the four groups of deep features which are fused by bagging ensemble learning. The feature extraction and ensemble learning mode are existing ones, so the innovation is limited.

**Response 1:**

The Transfer learning strategy used in this paper is freezing and training. When training four Convolutional neural network models, freeze part of the feature layers, retrain the rest of the feature layers and the full connection layer, and constantly adjust the strategy according to the performance on the food data set to obtain better results.

In this paper, a food image recognition method based on ensemble learning and transfer learning was proposed to detect nutritional contents in common foods. Six strategies were used on food-11 dataset for data augmentation and a large number of comparative experiments were carried out for food recogniton. The results of four CNN models on the Food-11 dataset demonstrated that the transfer learning method could effectively reduce the overfitting phenomenon and improve the generalization performance of the model. By combining different learning strategies, it could be proven that the ensemble learning method effectively improved the accuracy of the model, and the weight-ensemble method was the best for ensemble learning, achieving an accuracy rate of 96.88%. This demonstrated that the use of deep convolution neural network could make full use of the high-level semantic features of images, and the recognition method based on ensemble learning and transfer learning could help the model learn more comprehensive and rich feature information, greatly improving the generalization performance of the model. Therefore, the method proposed in this paper is suitable for food image recognition under complex background.

The next step of research will address the issue of reduced recognition speed caused by integration methods, streamline the structure of the integration model, optimize the integration methods, and achieve faster food image recognition speed.

**Point 2:** The authors need to explain the reasons why the four deep networks rather than other networks are chosen.

**Response 2:** When food image data with label information was not enough to train a complete deep convolutional neural network, in order to better complete the transfer learning task, VGG19, ResNet50, MobileNetV2, and AlexNet models were selected for experiments, which were all based on convolutional neural network, and had achieved great success in the ILSVRC (ImageNet large scale visual recognition challenge) competition. We have added relevant descriptions in section 3.2.

**Point 3:** Seen from the figure 2, the introduction of transfer learning is not apparent.

**Response 3:** We have replaced the figure 2.

**Point 4:** The sample size of the food images is smaller than that of the ImageNet, so simple fine tuning the the pretrained deep network is not enough. It is necessary to reduce the complexity of the network by pruning, in my opinion.

**Response 4:** I fully agree with your point of view, and this step is what we need to do next.

**Point 5:** Please explain how the parameter setup in the table 1 and table 3 is determined.

**Response 5:** The parameters in Table 1 refered to the papers of each model. The parameters in Table 3 were obtained through continuous experiments.

**Point 6:** Please explain why the deep networks in table 4 are chosen for comparison. Stand deviation of the accuracy is missing. In my opinion, the compared deep networks are old.

**Response 6:** We have added the column of standard deviation in Table 4. When food image data with label information was not enough to train a complete deep convolutional neural network, in order to better complete the transfer learning task, VGG19, ResNet50, MobileNetV2, and AlexNet models were selected for experiments, which were all based on convolutional neural network, and had achieved great success in the ILSVRC (ImageNet large scale visual recognition challenge) competition.

**Point 7:** Except the accuracy, other evaluation criteria are needed, such as AUC, F-score, confusion matrix, and so on.

**Response 7:** We have added the evaluation indicator F1-score in the paper.

**Point 8:** Ablation study is missing.

**Response 8:** The results of the ablation study were shown in table 4 and table 5.

**Point 9:** Complexity analysis is missing.

**Response 9:** We have added the evaluation indicator FPS and added relevant information in the paper.

Response to Reviewer 4 Comments

**Point 1:** The study on employing transfer learning and ensemble learning techniques for food recognition demonstrates significant contributions to the field. The methodology utilized, including the selection of appropriate pre-trained models and ensemble algorithms, showcases a thoughtful and effective approach to tackling the challenges in food recognition tasks.

**Response 1:** The review comments are good and do not require modification

Response to Reviewer 5 Comments

**Point 1:** Why choose VGG19, ResNet50, MobinetV2, and AlexNet in this study?

**Response 1:** When food image data with label information was not enough to train a complete deep convolutional neural network, in order to better complete the transfer learning task, VGG19, ResNet50, MobileNetV2, and AlexNet models were selected for experiments, which were all based on convolutional neural network, and had achieved great success in the ILSVRC (ImageNet large scale visual recognition challenge) competition. We have added relevant descriptions in section 3.2.

**Point 2:** Please clarify if the base learner refers to the architecture of the pre-trained model after freezing certain layers and retraining the remaining layers, while the probability distribution is denoted as hi(x). Additionally, in Figure 2, is the Output denoted as H(x)? Lastly, how are the values of wi in Eq (1) determined?

**Response 2:** We refered to the architecture of pre-trained models, froze certain layers

according to the performance and retrained the remaining layers.

The output was denoted as H(x).

We have added the description on how to calculate the value wi in the paper.

**Point 3:** Which evaluation indicator was used in this study as listed in Section 4.2?

**Response 3:** The evaluation indicators were all used in our research.

**Point 4:** It would be helpful if you could provide a list of the computation times for each experiment.

**Response 4:** We have added the evaluation indicator FPS(Frames per second).

**Point 5:** In Table 1, it appears that the number of layers in MobileNetV2 is incorrectly listed as 88. Could you please confirm that the correct number of layers is 53?

**Response 5:** We have corrected it.

**Point 6:** In Figure 6, please add in legend for the orange and blue lines.

**Response 6:** We have replaced the image.

**Point 7:** Write something below Section 2.

**Response 7:** We have added the relevant description.

Response to Reviewer 6 Comments

**Point 1:** Comparison with published research is required.

**Response 1:** We have added the relevant description in Section 1.

**Point 2:** Run-time analysis must be presented.

**Response 2:** We have added the evaluation indicator FPS in the paper.

**Point 3:** Authors are suggested to cite and discuss the following deep learning models.

‘**Deep learning: Applications, architectures, models, tools, and frameworks: A comprehensive survey**’,https://doi.org/10.1049/cit2.12180

‘Research on Face Intelligent Perception Technology Integrating Deep Learning under Different Illumination Intensities’ , https://doi.org/10.47852/bonviewJCCE19919

‘Deep transformer and few-shot learning for hyperspectral image classification’, https://doi.org/10.1049/cit2.12181

**Response 3:** We have cited the above papers and discussed it in our work.

**Point 4:** The details about parameters optimization is missing.

**Response 4:** We have added relevant information about parameter optimization in section 4.1.

**Point 5:** Manuscript requires correction of English.

**Response 5:** We have corrected it.
