# Supplementary material for: Recognition of food images based on transfer learning and ensemble learning
Source: PLoS One. 2024 Jan 19;19(1):e0296789. doi: 10.1371/journal.pone.0296789 (PMC10798480; doi:10.1371/journal.pone.0296789)
Supplement: Attachment — Submitted filename: Response to Reviewers.docx [file pone.0296789.s003.docx]

**Original Manuscript ID: PONE-D-23-15950R1**

**Original Article Title: “Recognition of Food Images Based on Transfer Learning and Ensemble Learning”**

**To:** Editor

**Re:** Response to reviewers

Dear Editor,

Thanks for your letter and review work on our manuscript entitled “Recognition of Food Images Based on Transfer Learning and Ensemble Learning”. We greatly appreciate the reviewers’ complimentary comments and suggestions. These comments are very helpful and valuable for improving and polishing our paper. The point-by-point responses to reviewers’ comments are given in the following part.

Best regards,

Le Bu, Caiping Hu, Xiuliang Zhang

**Reviews and Responses**

Point 1: Figure 1 was removed in the revised manuscript without tracked changes, but it remains in version with tracked changes. Which one is the correct version?

Response 1: In this version of the revised manuscript with tracked changes, we had removed the figure.

Point 2: In the with track changes manuscript, the revised Stage 1 in Figure 2 is misleading. It currently shows transfer learning from source dataset to target dataset, followed by data preprocessing. It should be revised to reflect the correct order: data preprocessing of the dataset, followed by transfer learning. Additionally, the figure does not align with the text explanation: "In the data preprocessing stage, the preprocessing operation of food images was mainly divided into 2 steps: data enhancement and data standardization." Please ensure that the text and figure match.

Response 2: We had replaced the figure to ensure the text and figure match.

Point 3: To improve clarity, please mention in text, Stage 1 represents preprocessing, Stage 2 is

the stage of pre-training network and fine-tuning the pre-training, and Stage is Ensemble

stage. Stage 2 should include 4 pre-trained models up to probability distribution, Stage 4

starts from Ensemble stage.

Response 3: We had replaced the figure.

Point 4: Please make sure that Stage 2 includes information up to Probability Distribution. Add in hi(x) for each Probability Distribution and H(x) at the Output.

Response 4: We had replaced the figure.

Point 5: Comment number 1 from Reviewer 6 should be added in the comparison of your results with other Food Image classification results in a table form in the Results section, not as the author claimed, already mentioned In Introduction part.

Response 5: We had added section 4.5.

Point 6: Comment number 4 from reviewer 6 has not been addressed at all, despite the authors’

claim that it has been mentioned in Section 4.1.

Comment number 4 from reviewer 6: The details about parameters optimization is missing.

Response 6: After our experiments, using the Adam optimizer could make the model converge faster, with initial learning rates set to 0.0001, training cycles of 150, and batch sizes of 64. And the different fine-tuning strategies adopted for each model were listed in section 4.1

Point 7: Please list the six augmentation strategies in the revised manuscript.

Response 7: The six augmentation strategies were listed in the second paragraph of section 3.1.

Point 8: The explanation of “The FPS indicator represents the time taken by the model to detect an image or the number of images processed in one second.” is misleading. If we use time taken by the model to detect an image, then the smaller FTS will be a good indicator. However, if we use “the number of images processed in one second “ then a larger FPS value is will be a good indicator. This leads to contradictory values for a good indicator. Please clarify this point.

Response 8: The FPS indicator represents the number of images processed in one second.

We had corrected relevant descriptions in section 4.2.

Point 9: The response to why the choice of the 4 pretrained models in Reviewers 3 and 5 should address why these four were chosen over others, not addressing the issue of limited images.

Response 9: Transfer learning first required pre-training CNN on a large-scale ImageNet dataset. The four network structures selected in this paper, AlexNet, Vgg19, MobileNet V2, and ResNet50, were all based on CNN and had certain representativeness. From AlexNet to ResNet50, the number of layers in the network continued to increase. VGG19 increased the nonlinear ability of the network, MobileNet V2 accelerated the computational speed of the model, and ResNet50 added cross layer connections to solve the problem of gradient vanishing. The depth of the network layer could be adjusted by increasing the number of convolutional layers, pooling layers, or fully connected layers. These four networks compensated for their respective shortcomings and facilitated the extraction of rich and complex high-dimensional image features, making them suitable for the food-11 dataset. Therefore, these four models were selected as base learners.

We had corrected relevant descriptions in section 3.2.
