# Supplementary material for: Recognition of food images based on transfer learning and ensemble learning
Source: PLoS One. 2024 Jan 19;19(1):e0296789. doi: 10.1371/journal.pone.0296789 (PMC10798480; doi:10.1371/journal.pone.0296789)
Supplement: Attachment — Submitted filename: Response to Reviewers.docx [file pone.0296789.s004.docx]

**Original Manuscript ID: PONE-D-23-15950R1**

**Original Article Title: “Recognition of Food Images Based on Transfer Learning and Ensemble Learning”**

**To:** Editor

**Re:** Response to reviewers

Dear Editor,

Thanks for your letter and review work on our manuscript entitled “Recognition of Food Images Based on Transfer Learning and Ensemble Learning”. In the previous revision, we mistakenly believed that the questions in the attachment included all the questions from Reviewer 5 and Reviewer 7, so we only answered the questions in the attachment. We greatly appreciate the reviewers’ complimentary comments and suggestions. These comments are very helpful and valuable for improving and polishing our paper. The point-by-point responses to Reviewer 7 comments are given in the following part.

Best regards,

Le Bu, Caiping Hu, Hui Sui, Xiuliang Zhang

**Reviews and Responses**

**Point 1:** In table 14, authors must cite the original research articles, currently only method name is there?

**Response 1:** There is no Table 14 in this article, I'm not sure if you're referring to Table 4. We temporarily understood it as the problem of Table 4. The citations of the original papers of different algorithms have been pointed out in the introduction part. The suffix "ft" indicated that this model underwent fine-tuning training under transfer learning methods, and different fine-tuning strategies have been pointed out in section 4.1.

**Point 2:** A comparison in terms of a large-scale dataset is required.

**Response 2:** We compared it to the food-101 dataset in section 4.5.

**Point 3:** Computational complexity is required to be discussed and presented in terms of Big-o-Notion.

**Response 3:** We have added relevant descriptions in section 4.4.

**Point 4:** How authors have selected research variables, it is not clear?

**Response 4:** The main research variables in the paper were the parameters of the different models. We referred to the original papers of different models and determined the model parameters based on a large number of experiments and research experience.

**Point 5:** Manuscript must be proof-read to fix grammatical errors.

**Response 5:** We have carefully checked the grammar of the paper and ensured that there are no errors. If you detect any errors, please help me point them out. We will be very grateful.
